# Improvement of Capillary Electrophoretic Enantioseparation of Fluoxetine by a Cationic Additive 

**Published:** 2013

**Authors:** Farin Sattary Javid, Alireza Shafaati, Afshin Zarghi

**Affiliations:** *Pharmaceutical Chemistry Department, School of Pharmacy, Shahid Beheshti University of Medical Sciences, Tehran, Iran. *

**Keywords:** Capillary electrophoresis, Chiral separation, Fluoxetine, Guanidine

## Abstract

One of the problems encountered in CE separations of basic compounds is the adsorption of analytes onto the negatively charged capillary wall which could lead to poor repeatability of migration time and peak area. Additionally, separation of enantiomers of chiral of basic drugs is commonly carried out in low pH buffer which contributes to strong ionic interaction of the cationic drug ions with negatively charged chiral selectors. The two phenomena results in poor enantioseparations. To overcome the problems associated with chiral separations of basic drugs by CE, the effect of guanidine (GU) on the improvement of chiral separation of a model basic drug, fluoxetine (FLX), was investigated. In the present study, GU was used as a cationic additive to the running buffer containing a chiral selector, sulfated beta cyclodextrine. Better results obtained with GU as the buffer additive in enantioseparation of FLX.

## Introduction

More than 30% of drugs in market are chiral (1) and mostly two enantiomers of a chiral drug show different pharmacological, toxicological or pharmacokinetic properties (2). 

In addition, separation methods are required for quality control of single-enantiomer pharmaceutical raw materials and products during their production, storage and application (3). Thus, different analytical techniques have been developed for controlling synthesis, enantiomer purity check and pharmacodynamic studies of chiral drugs. 

Capillary electrophoresis (CE) as a powerful technique, especially for analytical enantioseparations offers high efficiency and high flexibility with regard to the separation conditions, as well as the low consumption of chemicals and solvents. Consequently, it is shown that the major application of the technique is chiral separations (4). However, CE has some limitations, especially for chiral and non-chiral resolution of basic drugs. 

Basic analytes are positively charged over a wide range of pH (especially in acidic solutions) and electrostatically attracted to the negatively charged capillary walls. The irreproducible adsorption may cause variations to migration time and peak area (5), and consequently results in lower precision. Furthermore, impurity peaks may be masked by the peak tailing or resolution of enantiomers, in which two analytes migrating closely after each other may deteriorate. Additionally, analyte absorption could raise the limit of detection (LOD) and limit of quantification (LOQ) of analysis. 

Different approaches have been applied to avoid undesirable interactions of the analytes with the capillary wall, including the use of high salt concentration, extremes of pH, buffer additives, and coated capillaries (5). 

In the challenge of developing a separation method by capillary electrophoresis (CE), a variety of additives may be added to the background electrolyte to improve the separation (6) the most used additives are nonionic additive namely organic modifiers (7-8) and urea (9-10). The highly ionic triethylamine could optimize the resolution of separation of peptides (11). Our previous work (12) has shown that the addition of guanidine (GU) to the running buffer containing sulfated-b-cyclodextrin (SB-CD) as a chiral selector increased the resolution of enantiomers of basic chiral compounds. In the course of our ongoing interest in chiral separations, the present study was conducted to investigate the influence of GU on the SB-CD mediated enantioseparations of fluoxetine. 

**Figure 1:**
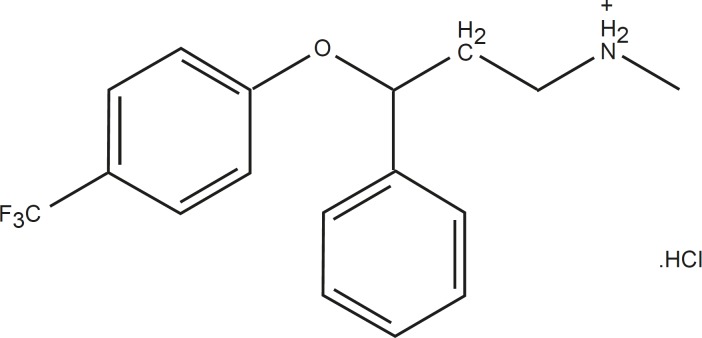
Chemical structure of fluoxetine HCl

Fluoxetine (FLX), as an antidepressant, is a selective serotonin reuptake inhibitor which is less sedating compared to tricylic antidepressants and has fewer antimuscarinic and cardiotoxic effects (13). FLX (Figure 1) is a basic drug which contains tertiary amine nitrogen (pK_a _of 10.3) (14) that is positively charged in neutral and lower pHs. This drug includes one chiral center within its structures. (*R*)-fluoxetine and (*S*)-fluoxetine are similarly effective at blocking serotonin reuptake. As these enantiomers are metabolized differently, the use of *R-*enantiomer was expected to result in less variable plasma levels of fluoxetine and its active metabolites compared to that observed with racemic fluoxetine. Additionally, it is shown that (*R*)- fluoxetine and its metabolites inhibit CYP2D6, a cytochrome P450 system enzyme, to a lesser extent than (*S*)-fluoxetine and its metabolites (15-16). 

Few reports on the application of CE methods were found in the literature for separation of FLX enantiomers (17-18). These methods presented the enantioseparation in acidic condition using combined neutral and negatively charged cyclodextrins. 

## Experimental

*Materials*

All solutions were prepared in Nanopure 18MΩ ultrapure water (Barnstead, Chicago, IL, USA). Sodium phosphate buffer with pH range of 2-4 was prepared from sodium monohydrogen phosphate purchased from Merck (Darmstadt, Germany), and adjusting the pH with orthophosphoric acid purchased from the same company, sulfated *β*-cyclodextrin (Sigma, Louis, MO, USA), were used as received. GU HCl was obtained from Merck (Darmstadt, Germany), Racemic FLX (as HCl) was kindly gifted by Noor Research and Education Institute (Tehran, Iran). 

*CE system*

All experiments were performed on a Prince-C650 CE system equipped with UV/ visible detector BISCHOFF (lambda 1010). CE analysis was performed using untreated fused-silica capillary of 50 μm internal diameter and 75-cm total length (25-cm effective length). The capillary was conditioned prior to the first use by rinsing with 1 M NaOH for 15 min, followed by 0.1 M NaOH and water for 5 min each. Between runs, the capillary was flushed for 3 min with running buffer to guarantee the good reproducibility. The electrophoretic integration was performed by Dax Data Acquisition Analysis software (version 8.0). 

*Capillary electrophoretic conditions*

The applied voltage was - 15 kV at 25°C, and the migrant species were detected at 230 nm. 

The running buffer was a 50 mM phosphate buffer adjusted at pH = 2-4 by 1 M sodium hydroxide. The running buffer contained 1-4% sulfated beta-cyclodextrine as chiral selector and guanidine (40-100 mM) as buffer additive, which was freshly prepared daily and filtered through a 0.45 μm filter membrane.

Sample injection was performed hydrodynamically by applying 50 mbar of pressure for 12 s.

*Sample preparations*

Standard stock solutions of racemic fluoxetine containing 1 mg/mL were prepared by dissolving standard powder in water. working solutions were prepared by diluting stock solution of fluoxetine with water.

## Results and Discussion

*Optimization of the method*

*Concentration of the selector*

**Figure 2:**
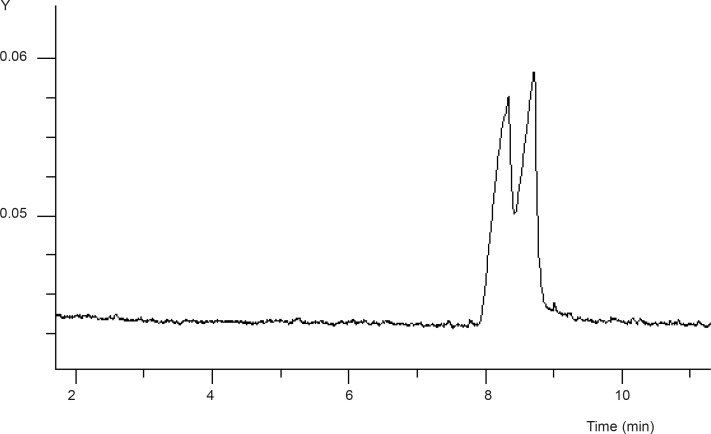
Separation of FLX enantiomers by CE. Experimental conditions: running buffer phosphate 25 mM (pH = 2.5) containing 3% SB-CD; wavelength, 230 nm; voltage, -15 kV; sample FLX 0.1 mg/mL, other condition as detailed in experimental section.

Based on our previous works on enantioseparation of basic drugs with CE (19-20), the initial attempt was based on applying a reversed-mode CE with the pH adjusted to 2.5 using a 25 mM phosphate buffer as the running buffer. The SB-CD concentration was studied over a range of 1-4% with maximum resolution achieved at 3% of SB-CD (see Figure 2). Lower concentrations of the selector resulted in very broad peak, whilst higher concentrations of the selector led to the deterioration of the enantioseparation.

*Concentration of the cationic additive*

**Figure 3:**
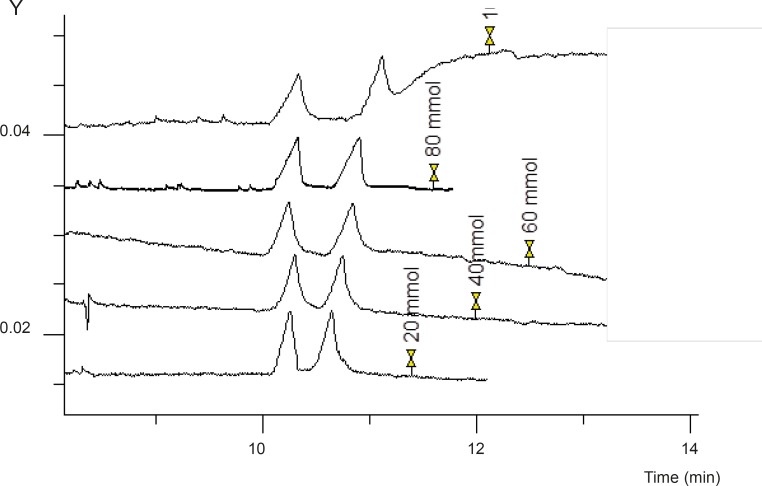
The effect of GU concentration on resolution of the two enantiomers of FLX Experimental conditions as described in Figure 2.

To improve chiral separation of the two enantiomers of FLX, different concentration of GU over a range of 20-100 mM was added to the running buffer. As shown in Figure 3, baseline separation of the enantiomers was obtained at any GU concentration. At higher GU concentrations, better peak shapes were observed. But to avoid Joule heating, GU concentration was adjusted to 80 mM which resulted in a reasonable separation and peak shapes, as well as minimum current and Joule heating.

*R* *unning buffer pH and concentration*

**Figure 4:**
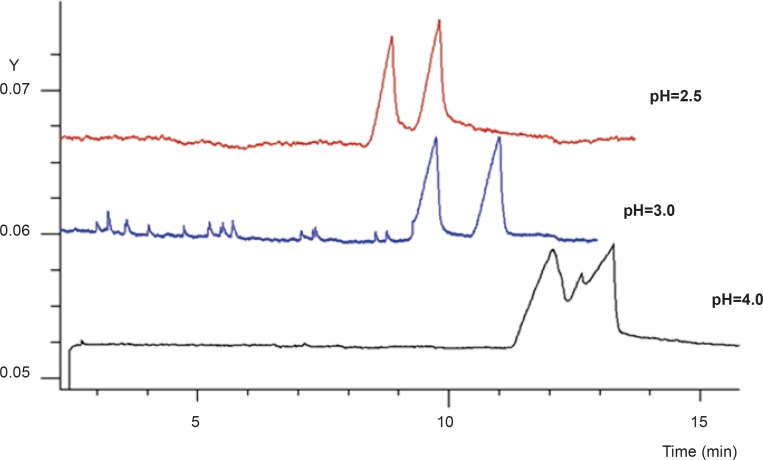
The effect of buffer pH on resolution of the two enantiomers of FLX Experimental conditions as described in Figure 2 (GU concentration was 80 mM).

Buffer pH is one of the most important factors in CE as it may affect the charge of the analytes and the chiral selector, and thus the binding characteristics (21). In order to obtain the optimum results, the buffer pH and concentration was reconsidered. Figure 4 shows the effect of buffer pH on the enantioseparation of FLX, with SB-CD and GU concentration kept constant at their best. Baseline resolution of the two peaks with acceptable peak shapes was observed at pH of 3. At this pH, the electroosmotic flow was low enough to allow applying carrier-mode separation, whilst ionization of FLX was high enough to render the formation of complex with negatively charged SB-CD molecules.

Despite better peak shapes obtained with higher buffer concentrations, to avoid Joule heating, buffer concentration was kept at 25 mM. Besides, GU was used as hydrochloride salt which in turn contributed to the buffer strength.

*Optimized resolution*

**Figure 5:**
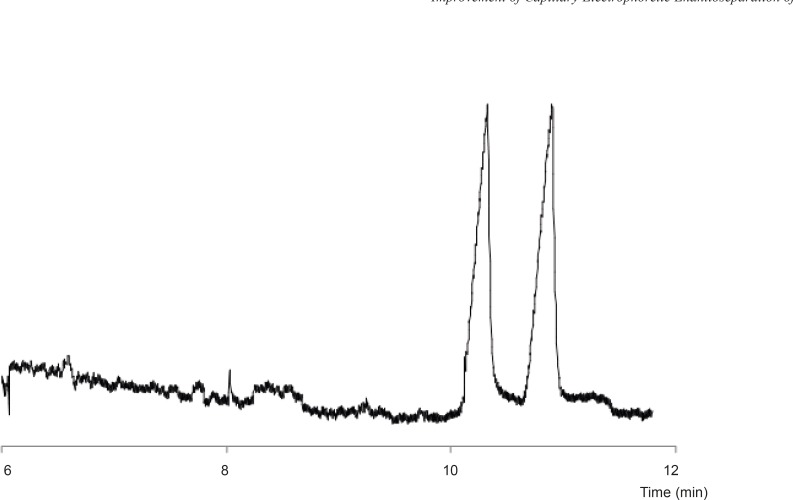
Resolution of the FLX enantiomers at optimum conditions.

Figure 5 shows the final electropherogram obtained at the optimum conditions, *i.e. *80 mM GU (as HCl) and 3% SB-CD in 25 mM phosphate buffer at pH of 3.0 with applied voltage of - 15 kV.

*GU Effects on enantioseparation*

The mechanism of chiral separation using CDs is based on the different inclusion of the hydrophobic part of the enantiomers into the CD cavity. The stereoselectivity is enhanced by secondary interactions between the functional groups of the analyte with the hydroxyl (or derivetized hydroxyl) groups on the outside rim of the CD (22). As shown in Figure 2, in the absence of GU, complete resolution of two enantiomers of FLX was not achieved which was attributed to the strong interaction of FLX cations with the sulfate groups on the CD molecule.

GU is a basic compound which bears positive charge in acidic solutions. The positive charge on the molecule is dispersed and delocalized (24). Thus, it is expected that the cation interacts with silanol groups on the inner surface of the bared capillary and prevents FLX cations to adsorb on the capillary wall, which contribute to the interaction of the drug and CD. In addition, it is suggested that GU modifies interaction between FLX cations and negatively charged SB-CD ions, which renders the inclusion of drug into the CD cavity (12).

## Conclusion

It was shown that GU greatly influenced resolution of the peaks relevant to the enantiomers of FLX when a reversed-mode CE method was applied using SB-CD in acidic buffer as a chiral selector. The GU effect was contributed to the modification of the interaction of FLX cations with the silanol groups on the capillary inner wall and with the negatively charged sulfate moieties on the CD molecules.
